# ﻿Novel gene rearrangement pattern in mitochondrial genome of *Ooencyrtusplautus* Huang & Noyes, 1994: new gene order in Encyrtidae (Hymenoptera, Chalcidoidea)

**DOI:** 10.3897/zookeys.1124.83811

**Published:** 2022-10-10

**Authors:** Zhi-Ping Xing, Xin Liang, Xu Wang, Hao-Yuan Hu, Yi-Xin Huang

**Affiliations:** 1 Collaborative Innovation Center of Recovery and Reconstruction of Degraded Ecosystem in Wanjiang Basin Co-founded by Anhui Province and Ministry of Education, Wuhu, Anhui 241000, China; 2 School of Ecology and Environment, Anhui Normal University, Wuhu, Anhui 241000, China; 3 Key Laboratory of Zoological Systematics and Evolution, Institute of Zoology, Chinese Academy of Sciences, 1 Beichen West Road, Chaoyang District, Beijing, 100101, China; 4 Anhui Provincial Key Laboratory of the Conservation and Exploitation of Biological Resources, College of Life Sciences, Anhui Normal University, Wuhu, Anhui 241000, China

**Keywords:** Encyrtinae, mitogenome, pairwise breakpoint distance, phylogenetic tree, Tetracneminae

## Abstract

Studies of mitochondrial genomes have a wide range of applications in phylogeny, population genetics, and evolutionary biology. In this study, we sequenced and analyzed the mitochondrial genome of *Ooencyrtusplautus* Huang & Noyes, 1994 (Hymenoptera, Encyrtidae). The nearly complete mitogenome of *O.plautus* was 15,730 bp in size, including 13 PCGs (protein-coding genes), 22 tRNAs, 2 rRNAs, and a nearly complete control region. The nucleotide composition was significantly biased toward adenine and thymine, with an A + T content of 84.6%. We used the reference sequence of *Chouioiacunea* and calculated the Ka/Ks ratio for each set of PCGs. The highest value of the Ka/Ks ratio within 13 PCGs was found in *nad2* with 1.1, suggesting that they were subjected to positive selection. This phenomenon was first discovered in Encyrtidae. Compared with other encyrtid mitogenomes, a translocation of *trnW* was found in *O.plautus*, which was the first of its kind to be reported in Encyrtidae. Comparing with ancestral arrangement pattern, wasps reflect extensive gene rearrangements. Although these insects have a high frequency of gene rearrangement, species from the same family and genus tend to have similar gene sequences. As the number of sequenced mitochondrial genomes in Chalcidoidea increases, we summarize some of the rules of gene rearrangement in Chalcidoidea, that is four gene clusters with frequent gene rearrangements. Ten mitogenomes were included to reconstruct the phylogenetic trees of Encyrtidae based on both 13 PCGs (nucleotides of protein coding genes) and AA matrix (amino acids of protein coding genes) using the maximum likelihood and Bayesian inference methods. The phylogenetic tree reconstructed by Bayesian inference based on AA data set showed that *Aenasiusarizonensis* and *Metaphycuseriococci* formed a clade representing Tetracneminae. The remaining six species formed a monophyletic clade representing Encyrtinae. In Encyrtinae, *Encyrtus* forms a monophyletic clade as a sister group to the clade formed by *O.plautus* and *Diaphorencyrtusaligarhensis. Encyrtussasakii* and *Encyrtusrhodooccisiae* were most closely related species in this monophyletic clade. In addition, gene rearrangements can provide a valuable information for molecular phylogenetic reconstruction. These results enhance our understanding of phylogenetic relationships among Encyrtidae.

## ﻿Introduction

**Figure 1.**
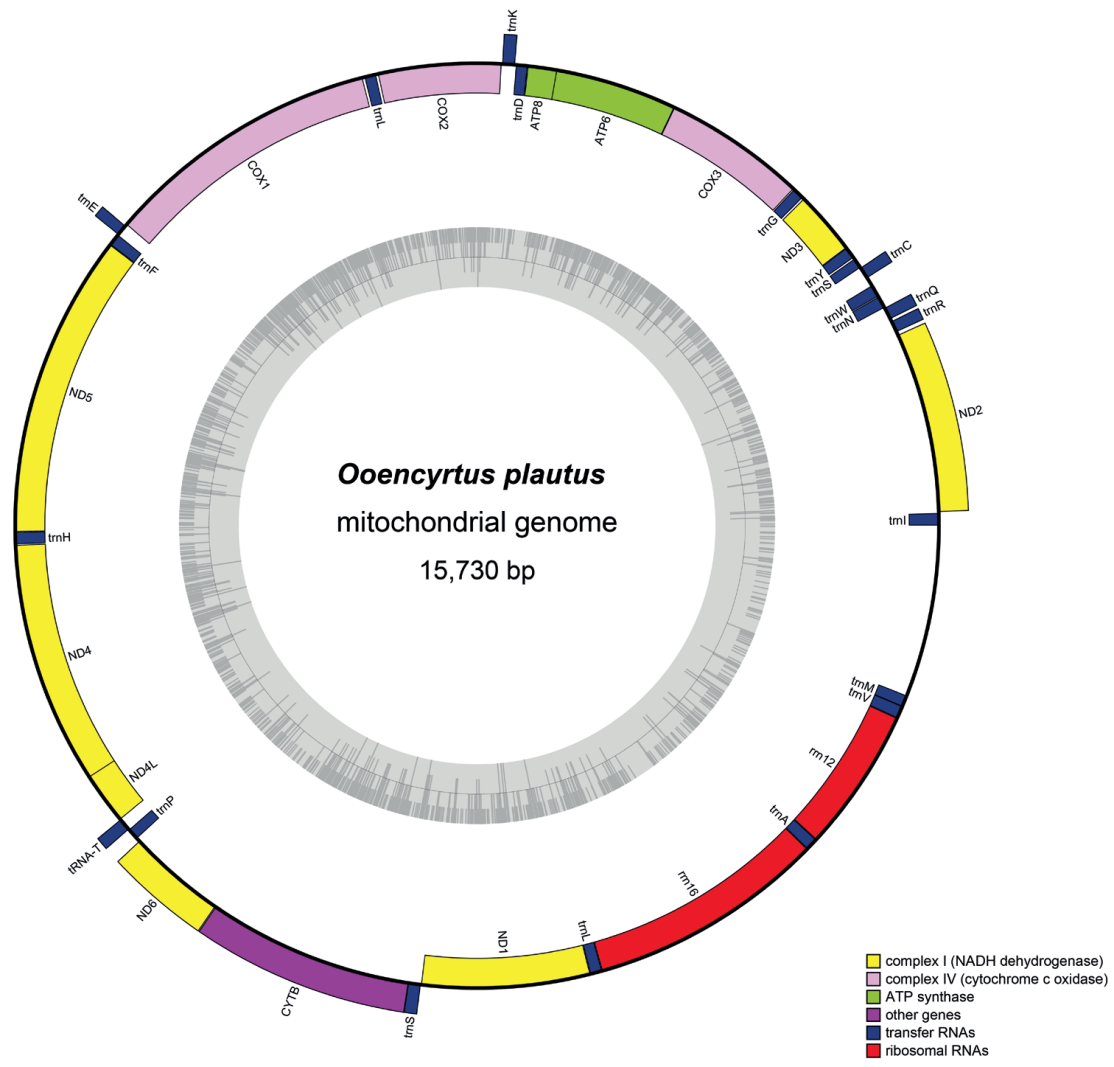
Circular map of the *Ooencyrtusplautus* mitochondrial genome.

The mitochondrial genome is a standard circular molecule, mostly range between 15 kb and 18 kb in size, with 37 genes, including 13 protein-coding genes, three of which are oxidative phosphorylation complexes, 2 rRNAs, and 22 tRNAs and a major non-coding region which mainly regulates replication and transcription (Fig. 1) (Boore 1999; Cameron 2014). The mitochondrial (mt) genome has the characteristics of gene recombination, maternal inheritance, conservation of gene components, and high AT content, and it is considered to be an ideal molecular marker for species identification and phylogenetic or evolutionary studies (Boore and Brown 1998; Curole and Kocher 1999). In addition, the gene content, genome size, and RNA secondary structure of the mt genome can also provide useful information for phylogeny (Boore and Brown 1998; Gissi et al. 2008; Cameron 2014). Gene rearrangement is one of the most frequently studied features in animal mitochondrial genomes (Boore and Brown 1998; Cameron 2014; Li et al. 2016). It is usually conserved in major lineages but may be rearranged in some groups (Boore and Brown 1998; Boore 1999; Dowton et al. 2002a). The rearrangement processes of mitochondrial genes can be described as transposition, inversion, reverse transposition, and TDRL (tandem-duplication-random-loss) (Dowton et al. 2002b; Cameron 2014). The movement of TDRL is to describe the duplication of multiple consecutive genes and the successive random loss of one of the two copies (Bernt et al. 2007). Previous works by Sankoff et al. (1992) and Boore et al. (1998) showed that the gene sequence of the mitochondrial genome contains phylogenetic signals. Studying gene rearrangements in lower taxonomic lineages of insects can provide some evidence for the evolution of these groups (Mao et al. 2014; Li et al. 2016).

**Figure 2.**
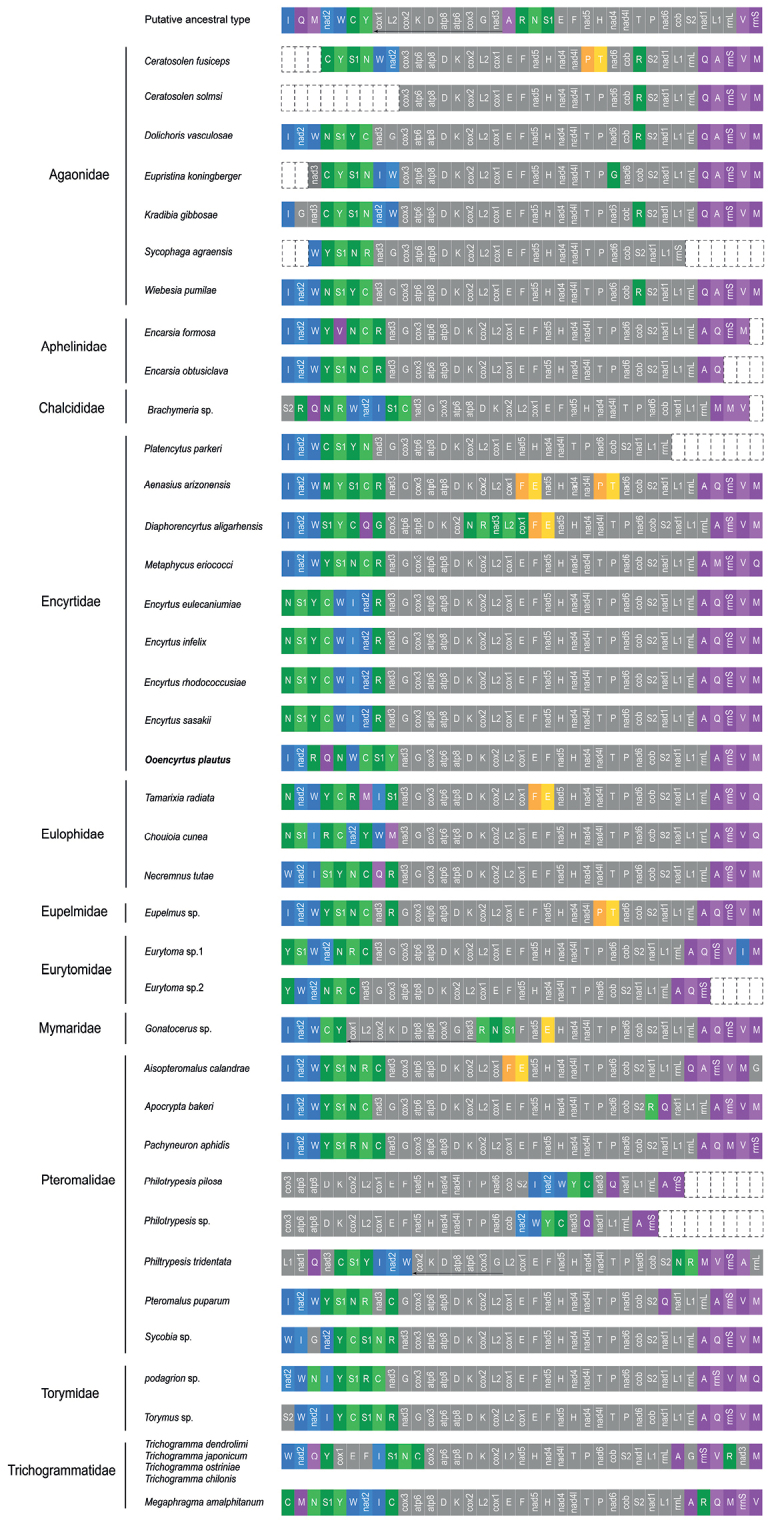
Mitochondrial genome organization in Chalcidoidea. Colored boxes indicate rearranged gene clusters, and gray boxes indicate conserved gene blocks.

Extensive mitochondrial genome data indicates that, compared to other orders in the hexapoda, Hymenoptera (Dowton and Austin 1999; Dowton et al. 2009; Wei et al. 2014) and Hemiptera (Shao and Barker 2003; Shao et al. 2003; Johnson et al. 2004) have highly accelerated and independent gene arrangement events. Chalcidoidea is one of the most diverse groups of insects in the order Hymenoptera. Parasitic chalcidoids are widely used as biological control of various agricultural pests (Heraty 2017). At the same time, diverse morphology, body sizes, lifestyles, and different types of parasitism, which is also reflected in their amazing genetic arrangement (Austin et al. 1998; Heraty et al. 2013). The mitochondrial genome of Chalcidoidea has been confirmed to have a large number of gene rearrangements (Oliveira et al. 2008; Xiao et al. 2011). A total of 40 species of mitochondrial genomes have been reported in the Chalcidoidea, including 11 families and all sequenced mitochondrial genomes have rearrangements compared with putative ancestor (Fig. 2) (up to date: 15 March 2022). The parasitic life history, body size, and developmental period in Hymenoptera are hypothesized to be related to their gene rearrangements (Dowton and Austin 1995; Shao et al. 2001), and the information of these gene rearrangements may be valuable for the phylogenetic reconstruction of specific lineages (Yuan et al. 2015; Liu et al. 2017).

*Ooencyrtusplautus* is a kind of parasitoid in the family Encyrtidae, which has the characteristics of high parasitism rate and strong reproduction ability. In the Encyrtidae, seven species of complete mitochondrial genes and one species of partial mitochondrial genes have been reported (Table 1). In this study, the nearly complete mitochondrial genome of *O.plautus* was measured, sequenced, and annotated. The characteristics of the mitogenomes are described in terms of genome structure, nucleotide content, frequency of usage of start and stop codons, and gene rearrangement. It reported a new gene rearrangement which was found in the mitochondrial genome of *O.plautus* (The long-distance transposition of *trnW* and the reverse transposition of gene cluster *trnN*-*trnS1*-*trnY*) and conducted phylogenetic analyses using the current mitogenome along with those previously reported from Encyrtidae.

**Table 1.** List of species investigated and their related information.

	Family	Subfamily	Taxa	GenBank Accession No.	Location/Refence
1	Aphelinidae		*Encarsiaformosa*	MG813797.1	(Zhu et al. 2018)
2			*Encarsiaobtusiclava*	MG813798.1	(Zhu et al. 2018)
3	Encyrtidae	Tetracneminae	*Aenasiusarizonensis*	MK630013	(Ma et al. 2019)
4		Tetracneminae	*Metaphycuseriococci*	MW255970	Direct Submission
5		Encyrtinae	*Diaphorencyrtusaligarhensis*	MN274569	(Du et al. 2019)
6		Encyrtinae	*Encyrtuseulecaniumiae*	NC_051459	Direct Submission
7		Encyrtinae	*Encyrtusinfelix*	MH729198	(Xiong et al. 2019)
8		Encyrtinae	*Encytusrhodooccisiae*	NC_051460	Direct Submission
9		Encyrtinae	*Encyrtussasakii*	NC_051458	Direct Submission
10		Encyrtinae	*Ooencyrtusplautus*	OP442361	This study

## ﻿Materials and methods

### ﻿Sample collection and DNA extraction

Specimens of *Ooencyrtusplautus* were collected from Fuzhou city, Fujian province, in September 2020. They were reared in the laboratory, then processed for DNA extraction. Total genomic DNA was extracted using the cetyltrimethyl ammonium bromide (CTAB) method (Shahjahan et al. 1995).

### ﻿Sequencing and assembly

Sequencing was performed using a whole genome shotgun (WGS) strategy on the Illumina Novaseq platform. The quality of data was checked using FastQC (Andrews 2013). Raw data were filtered into high-quality data after processing with AdapterRemoval v. 2. Then, Single Nucleotide Polymorphism (SNP), Insertion and Deletion (InDel), Copy Number Variation (CNV), and Structural Variation (SV) analysis were conducted to ensure the reliability of nucleotides. The assembly of the mitochondrial genome was accomplished with Novoplasy v. 2.7 (Dierckxsens et al. 2017).

### ﻿Mitochondrial genome annotation

Gene annotation of mitochondrial genome was performed using MitoZ and 13 protein-coding genes as well as 22 tRNA genes are annotated (Meng et al. 2019). Twenty-two tRNA genes were verified with the use of MITOS WebServer, setting the parameters with the Invertebrate Mito genetic code (Minh et al. 2013). Every sequence of tRNA genes was manually checked separately. According to the secondary structure of tRNA predicted by MITOS, it was drawn manually using Adobe Illustrator. Annotation of the 13 protein-coding genes were refined manually by identifying the corresponding open reading frames using the invertebrate mitochondrial code. The mitogenome maps were produced using Organellar Genome DRAW (OGDRAW) (Marc et al. 2013).

### ﻿Comparative analysis

Geneious v. 11.0.2 was used to examine all genes in the mitochondrial genome (Kearse et al. 2012). MEGA X (Sudhir et al. 2018) was used to analyze base composition and relative synonymous codon usage (RSCU). To calculate the number of synonymous substitutions for each synonymous site (Ks) and the number of non-synonymous substitutions for each non-synonymous site (Ka) through DnaSP 5. AT/GC skewness is calculated as AT skewness = (A − T) / (A + T) and GC-skew = (G − C) / (G + C) (Rozas et al. 2003). To measure the relative composition of different bases was measured based on GC and AT skew.

### ﻿Phylogenetic analysis

In this study, a total of 10 species of mitogenomes were analyzed, of which *Encarsiaformosa* and *Encarsiaobtusiclava* of family Aphelinidae were selected as outgroups (Table 1). MAFFT v. 7.3.1 was used to align the PCGs (Kazutaka and Standley 2016). Alignments of individual genes were concatenated to generate two kind of data sets: 1) the PCG matrix, including all three codon positions of protein-coding genes; 2) the AA matrix, translated 13 PCG into amino acids.

Both ML (maximum likelihood) and BI (Bayesian inference) analyses were performed on the concatenated data set used for phylogenetic reconstruction. In W-IQtree (Trifinopoulos et al. 2016), the best-fit model was used for maximum likelihood analysis. The site-heterogeneous mixture model (CAR+GTR) was used in PhyloBayes analysis. The trees were sampled every 1000 generations (Huelsenbeck 2012). FigTree v. 1.3.1 was used to view the generated tree.

## ﻿Results

### ﻿Genome structure and organization

**Figure 3.**
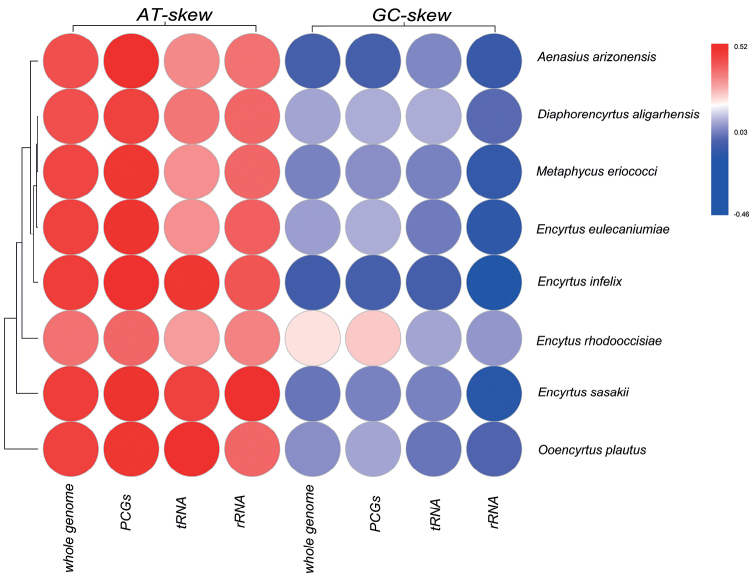
Nucleotide composition of various data sets of mitogenomes. Hierarchical clustering of Encyrtidae species based on the AT-skew and GC-skew.

The nearly complete mitogenome of *O.plautus* was 15,730 bp in size, including 13 PCGs, 22 tRNAs, 2 rRNAs, and a nearly complete control region. In *Ooencyrtusplautus*, 27 genes (15 tRNAs, 2 rRNAs, and 10 PCGs) were encoded by the majority-strand (J-strand), and 10 genes (3 PCGs and 7 tRNAs) were encoded by the minority-strand (N-strand). The values of AT skew and GC skew are often used to reveal the nucleotide composition of the mitochondrial genome (Alexandre et al. 2005). In this study, the nucleotide compositions of eight complete or nearly complete mitogenomes in Encyrtidae were investigated by calculating the percentages of AT-skew and GC-skew (Fig. 3). The results of the nucleotide skew statistics showed that the AT-skews in PCGs, tRNAs, and rRNAs of encyrtid mitogenomes were almost all positive, while the GC-skews were almost all obviously negative.

The nucleotide composition *O.plautus* was significantly biased toward adenine and thymine, with an A + T content of 84.6% (Table 2). The PCGs on the N strand had a T skew and slight G skew. However, the PCGs on the J strand exhibited a C skew. The tRNAs was negative for AT skews, besides the tRNAs on the N strand, which had an equal G and C distribution. Two rRNA genes (lrRNA and srRNA) were encoded on the J strand and exhibit an A skew and slight C skew.

**Table 2.** Nucleotide composition and skewing of the *Ooencyrtusplautus* mitogenome.

	size	T	C	A	G	AT-skew	GC-skew
whole genome	15730	0.401	0.085	0.445	0.069	0.052	-0.104
PCGs	11072	0.381	0.097	0.439	0.083	0.071	-0.078
PCGs(J)	8402	0.345	0.086	0.467	0.102	0.150	0.085
PCGs(N)	2670	0.494	0.081	0.352	0.073	-0.168	-0.052
tRNA	1467	0.455	0.066	0.437	0.042	-0.020	-0.222
tRNA(J)	994	0.454	0.036	0.437	0.073	-0.019	0.339
tRNA(N)	473	0.457	0.053	0.438	0.053	-0.021	0.000
rRNA	2103	0.437	0.068	0.453	0.042	0.018	-0.236

Intergenic spacers and overlapping genes are very common in arthropod mitochondrial genomes (Ma et al. 2015; Chen et al. 2018). In the mitochondrial genome of *O.plautus*, a total of 166 bp of intergenic spacers ranging from 1 to 47 bp were found in eight locations. The minimum intergenic spacers (1 bp) located at *trnT*-*trnP*, *trnQ*-*trnW*, *nad6*-*cob*, and *trnS2*-*nd1*. The maximum (47 bp) gene spacer was located at *trnW*-*trnC*. There were 18 overlapping gene regions, ranging from 2 bp to 7 bp in length in the nine mitogenomes. The longest overlapping sequence (7 bp) was between *atp6*-*atp8* and *nad4*-*nad4l*.

### ﻿Protein-coding genes (PCGs)

In the mitochondrial genome of *O.plautus*, the total length of protein-coding genes was 11,072 bp, accounting for 70.39% of the entire genome. Most of them were encoded on the J strand, and only *nad2*, *nad6*, and *cob* were encoded on the N strand. The average A + T content of the 13 protein-coding genes was 82%, ranging from 73.7% (*cox1*) to 93.80% (*atp8*) for individual genes.

**Figure 4.**
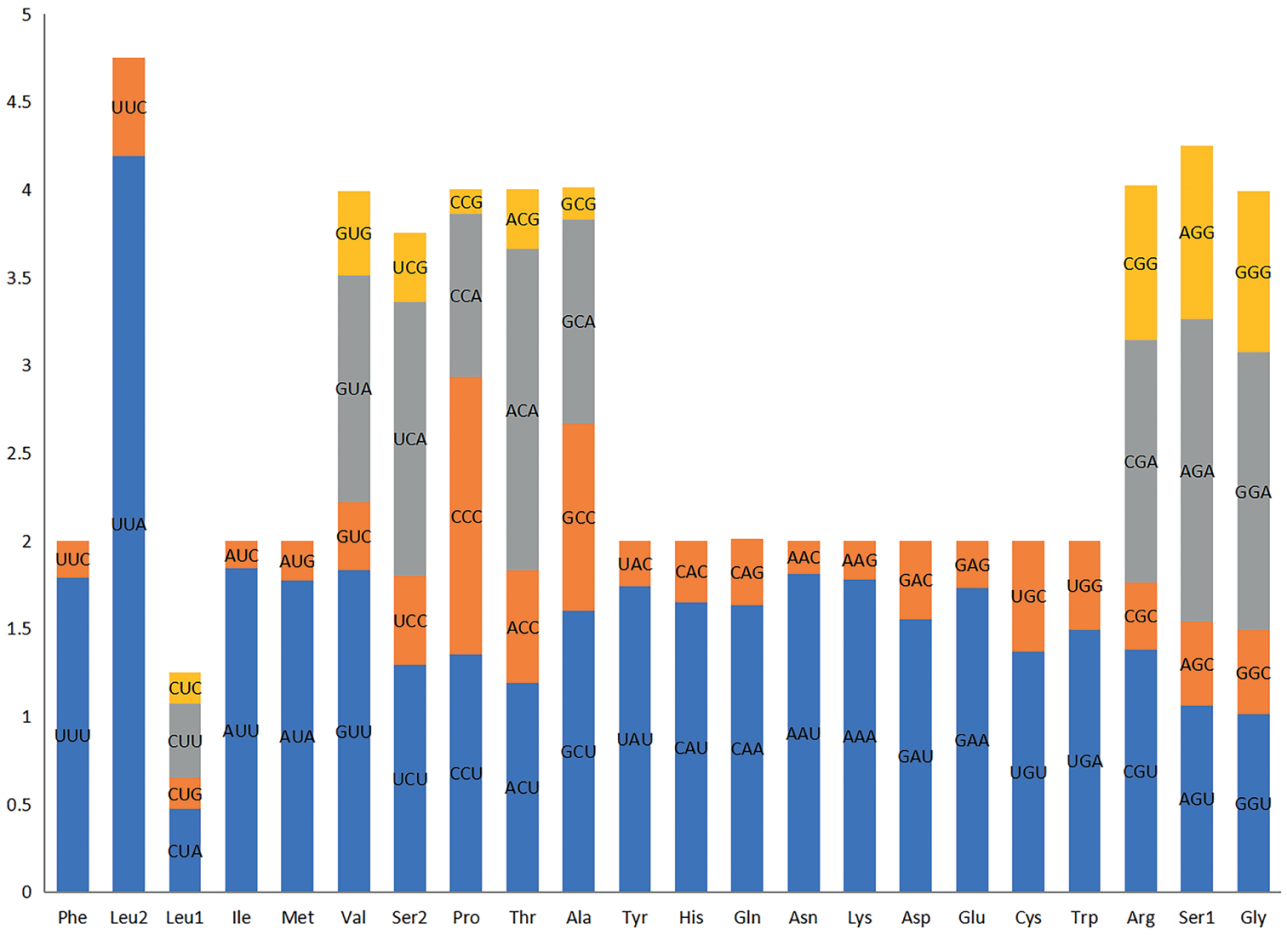
Relative synonymous codon usage (RSCU) of the mitochondrial genomes of *Ooencyrtusplautus*.

To investigate further this high A and T content, and the frequency of synonymous codon usage, we calculated relative synonymous codon usage (RSCU) values. The relative synonymous codon usages (RSCU) of the *O.plautus* are shown in Fig. 4. Taken together, the most frequently used codons are UUA (Leu2), AGA (Ser1), and CGA (Arg), whereas those ending in G or C, CUG, CUC, CAG, and GGC were the less frequently used codons. The codons ending with A or T are predominant, which at least partly leads to the bias towards A and T.

The predicted initiation codons are ATN as in most other insect mitochondrial genomes (Dowton and Austin 1999). There were six genes (*cox1*, *cob*, *nad4*, *nad6*, *atp6*, *and cox3*) starting with ATG and seven genes (*nad1*, *nad3*, *nad6*, *nad4L*, *nad5*, *cox2*, *and atp8*) starting with ATT. All protein-coding genes terminated at the most common stop codon TAA, except for *nad1* and *nad5*, which stopped with single T (Table 3).

**Table 3.** Mitogenomic organization of *Ooencyrtusplautus*.

Name	Start	Stop	Strand	Length	Codons
*trnI*	1	68	+	68	
*nad2*	1064	75	-	870	ATT/TAA
*trnR*	1146	1082	-	65	
*trnQ*	1227	1160	-	68	
*trnN*	1229	1296	+	68	
*trnW*	1299	1367	+	69	
*trnC*	1481	1415	-	67	
*trnS1*	1482	1540	+	59	
*trnY*	1548	1613	+	66	
*nad3*	1613	1966	+	333	ATT/TAA
*trnG*	1975	2039	+	65	
*cox3*	2043	2828	+	747	ATG/TAA
*atp6*	2828	3502	+	666	ATG/TAA
*atp8*	3496	3657	+	159	ATT/TAA
*trnD*	3658	3721	+	64	
*trnK*	3796	3727	-	70	
*cox2*	3800	4474	+	639	ATT/TAA
*trnL2*	4486	4549	+	64	
*cox1*	4561	6090	+	1509	ATG/TAA
*trnE*	6161	6099	-	63	
*trnF*	6162	6229	+	68	
*nad5*	6230	7898	+	1419	ATT/T
*trnH*	7899	7966	+	68	
*nad4*	7970	9307	+	1281	ATG/TAA
*nad4l*	9301	9588	+	255	ATT/TAA
*trnT*	9589	9659	-	71	
*trnP*	9661	9725	+	65	
*nad6*	10289	9744	-	501	ATG/TAA
*cob*	11424	10291	-	1098	ATG/TAA
*trnS2*	11491	11423	-	69	
*nad1*	11493	12417	+	906	ATT/T
*trnL1*	12418	12483	+	66	
*rrnL*	12484	13791	+	1308	
*trnA*	13792	13860	+	69	
*rrnS*	13861	14655	+	795	
*trnV*	14656	14725	+	70	
*trnM*	14724	14788	+	65	

**Figure 5.**
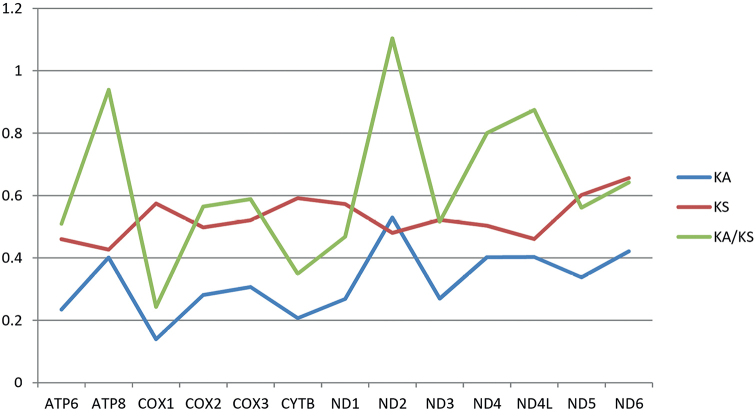
Evolutionary rates of mitochondrial genomes in *Ooencyrtusplautus*. The numbers of nonsynonymous substitutions per nonsynonymous site (Ka), the number of substitutions per synonymous site (Ks), and the ratio of Ka/Ks for each gene are given, using *Chouioiacunea* as the reference sequence.

Previous work (Zhang et al. 2020) showed that Eulophidae and Encyrtidae have close relationships, so we chose Eulophidae species as the reference sequence. Using *Chouioiacunea* as a reference sequence, we calculated the non-synonymous substitution rate (Ka), synonymous substitution (Ks), and Ka/Ks ratio of each PCG for *O.plautus* (Fig. 5). Ka and Ks are non-synonymous and synonymous substitution rates, respectively. They are controlled by functionally related sequence contexts, such as encoding amino acids and participating in exon splicing (Parmley and Hurst 2007). The ratio of the two parameters Ka/Ks (a measure of the strength of selection) is defined as the degree of evolutionary change (Wang et al. 2011). A value of Ka/Ks greater than 1 means positive selection exists, indicating that non-synonymous mutations are more favored by Darwinian selection, and they will be retained at a rate greater than synonymous mutations. In *O.plautus*, the Ka/Ks ratio of *nad2* was greater than 1, indicating that the *nad2* gene had a positive selection. This phenomenon was first discovered in Encyrtidae. In addition, the Ka/Ks of *atp8* is as high as 0.9389, which is the highest except for *nad2*. The high Ka/Ks phenomenon of *nad2* and *atp8* is also found in other species (Jia et al. 2020; Guo et al. 2021; Xu et al. 2021). The reason for this phenomenon may be that the evolution speed of a gene is related to its function (Wang et al. 2011).

### ﻿Transfer and ribosomal RNA genes

In total, 22 transfer RNA genes were found, ranging in size from 59 bp (*trnS1*) to 71 bp (*trnT*). Most of the tRNAs were encoded on the J strand and only 7 tRNAs (*trnT*, *trnE*, *trnK*, *trnC*, *trnQ*, *trnR*, *and trnS2*) were encoded on the N strand. The average nucleotide composition of these tRNAs was A: 43.7%, T: 45.5%, C: 6.6%, and G: 4.2%, with a total average A + T content of 89.2%. All tRNA sequences can be folded into the canonical cloverleaf secondary structure, except for *trnS1* which lacked the dihydrouridine (DHU) arm. A lack of the DHU arm in *trnS1* was found in the mitochondrial genomes of most insects (Dowton et al. 2002b). Changes in the length of the DHU and TΨC arms lead to differences in the size of the tRNA sequence (Shao et al. 2001). In addition, some mismatches (G-U in *trnD*, *trnA*, and *trnV*, U-U in *trnS1*, and two U-U in *trnA*) were found in *O.plautus*.

The two rRNA genes, the larger ribosomal gene (rrnL) and the smaller ribosomal gene (rrnS), were located between *trnA* and *trnL1*, and *trnV* and *trnA*, respectively. The average of the total size of two rRNAs was 2,103 bp and the average A + T content was 89%.

**Figure 6.**
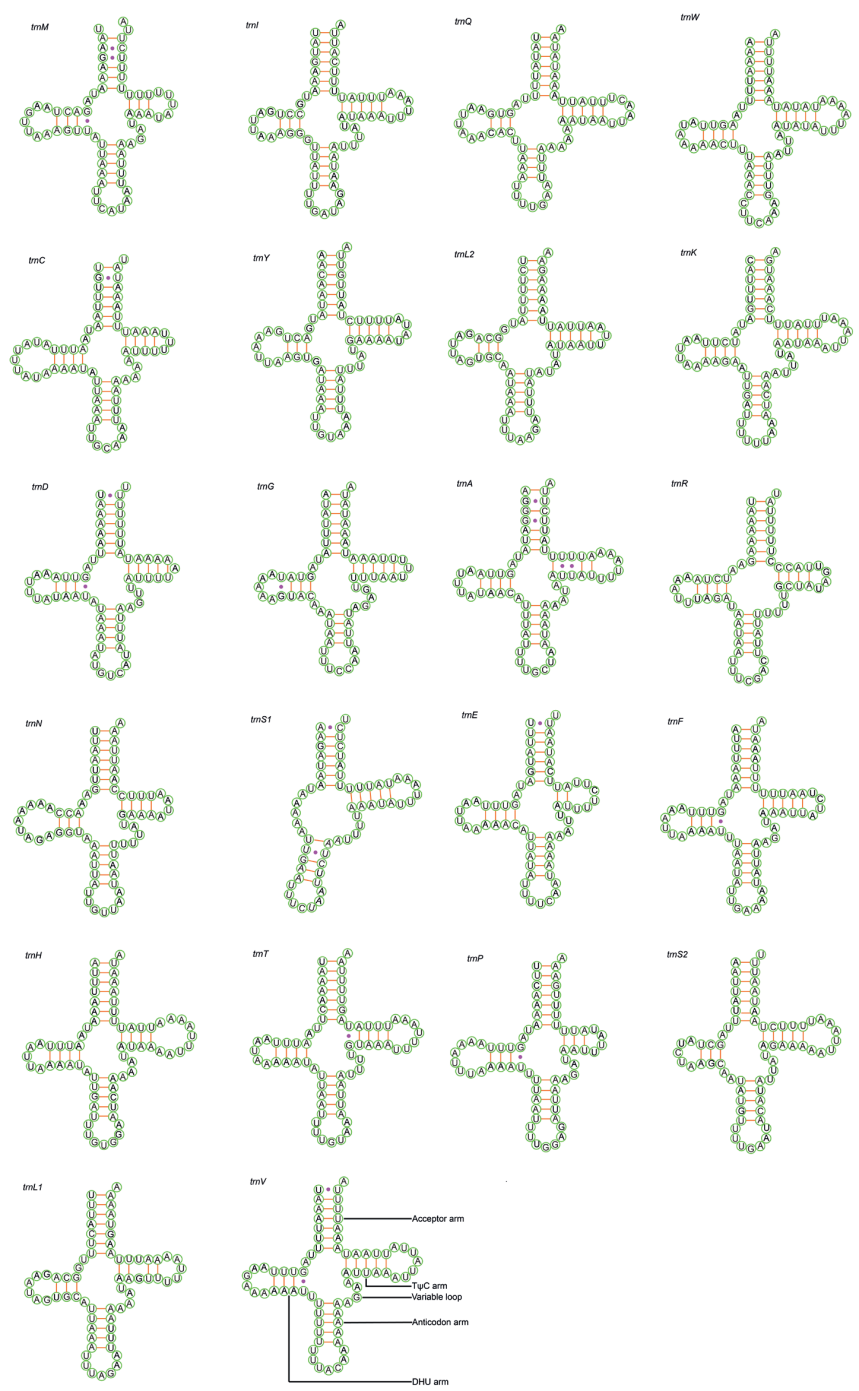
Predicted secondary cloverleaf structure for the tRNAs of *Ooencyrtusplautus*.

### ﻿Gene arrangement patterns

**Figure 7.**
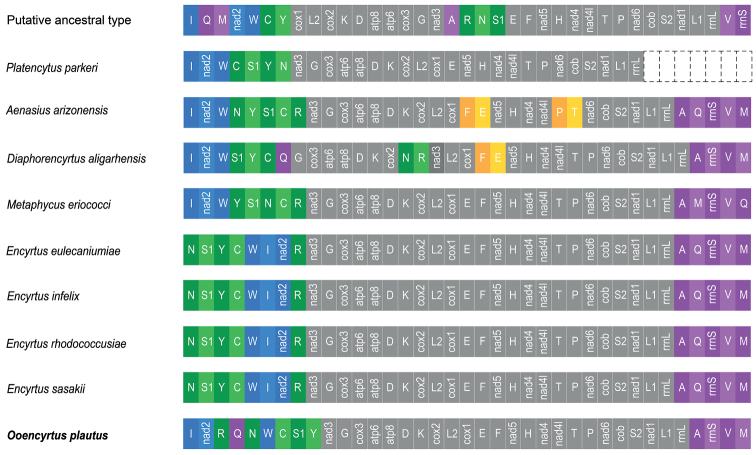
Mitochondrial genome organization in Encyrtidae. Colored boxes indicate rearranged gene clusters, and gray boxes indicate conserved gene blocks.

Gene rearrangement in the mitochondrial genome is a relatively rare event in the evolutionary history of insects (Cameron 2014). However, in the Hymenoptera lineage, more and more species have mitochondrial gene rearrangements (Shao et al. 2003). Gene rearrangements are a common phenomenon found in almost all the sequenced Chalcidoideamt genomes (Fig. 2). In the Chalcidoidea mitochondrial genomes studies, most of the tRNA genes are rearranged (Tang et al. 2018). In the Hymenoptera, numerous rearrangements of protein-coding genes have been identified in several groups (Xiao et al. 2011; Wei et al. 2014). Compared with the putative ancestral pattern of the insect mitochondrial genome, dramatic gene rearrangements, not only in tRNA genes but also in protein-coding genes, were found in *O.plautus* mitochondrial genomes. In the mitogenome of *O.plautus*, a total of 17 genes, including six PCGs, 10 tRNAs, and one rRNA gene are rearranged (Fig. 7).

In Encyrtidae, the gene order of all species of *Encytus* are identical. We used their gene order as a template to analyze the gene rearrangement of Encyrtidae. It can be summarized into four obvious rearrangements. They are the rearrangement of gene clusters *trnI-nad2-trnW*, *trnY-trnS1-trnN-trnC-trnR*, and *trnA-trnQ-rrns-trnV-trnM*. In addition, there are inversions of *trnE-trnF* and *trnP-trnT*.

Firstly, the *trnW-trnI-nad2* gene cluster in these four species (*Aenasiusarizonensis*, *Diaphorencyrtusaligarhensis*, *Metaphycuseriococci*, and *Platencytusparkeri*) was rearranged as *trnI-nad2-trnW*, most species in Chalcidoidea of which were in this order. However, in *O.plautus*, *trnW* was translocated, causing the gene cluster *trnI-nad2-trnW* to be divided into *trnI-nad2* and *trnW*, which is the first of its kind to be reported in Chalcidoidea. Besides, the *trnI-nad2* gene cluster was translocated to upstream. Secondly, the *trnN-trnS1-trnY-trnC-trnR* gene clusters in four species (*A.arizonensis*, *D.aligarhensis*, *M.eriococci*, and *O.plautus*) have undergone disorderly rearrangements. Thirdly, *trnE-trnF* inversion occurred in both *A.arizonensis* and *D.aligarhensis*. In addition, *trnP-trnT* inversion also occurred in *D.aligarhensis*. Finally, in *D.aligarhensis* and *O.plautus*, *trnQ* in the gene cluster *trnA-trnQ-rrnS-trnV-trnM* has a long-distance transposition. In *M.eriococci*, *trnM* and *trnQ* were transposed in the gene cluster *trnA-trnQ-rrnS-trnV-trnM*.

### ﻿Phylogenetic analyses

Phylogenetic relationships were analyzed using the concatenated nucleotides and amino acids sequences of 13 PCGs from eight encyrtid species and two outgroups. Four topologies were constructed using both ML and BI methods and two different data sets.

**Figure 8.**
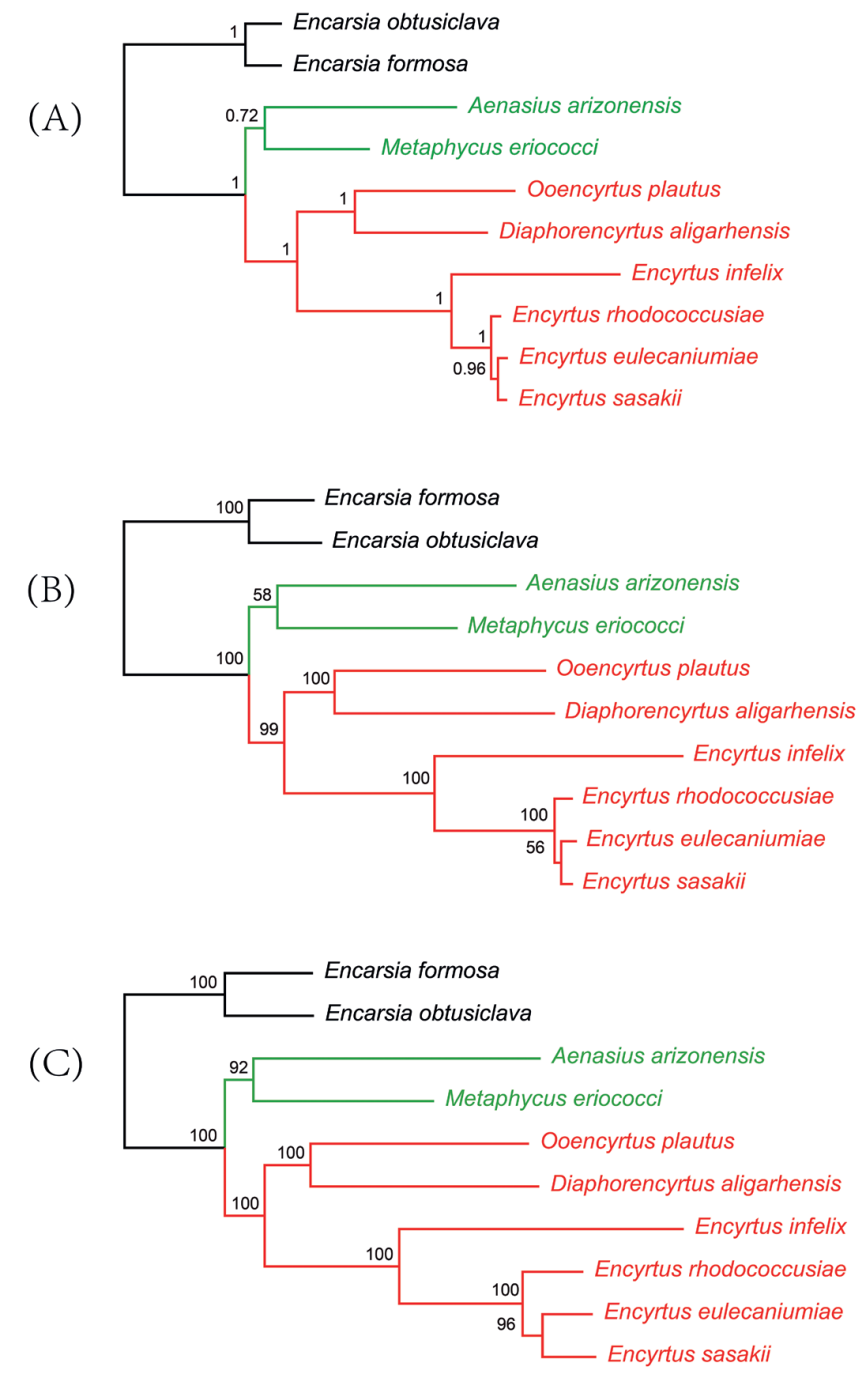
Phylogenetic tree produced by maximum likelihood and Bayesian inference analyses based on the 13 PCG and the AA data set **A** phylogenetic tree produced by Bayesian inference analyses based on 13 PCG data set **B** phylogenetic tree produced by maximum likelihood analyses based on the AA data set **C** phylogenetic tree produced by maximum likelihood analyses based on the 13 PCG data set.

The topological structures of these two phylogenetic trees reconstructed by ML analysis were identical with the phylogenetic tree reconstructed by BI based on PCG (Fig. 8). While Bayesian tree reconstructed on the AA data set showed different phylogenetic relationships in the clade consist of *Encyrtusrhodococcusiae*, *E.eulecaniumiae* and *E.sasakii* which was *E.eulecaniumiae* + (*E.rhodococcusiae + E.sasakii*) rather than *E.rhodococcusiae* + (*E.eulecaniumiae + E.sasakii*). The deviation and rate heterogeneity of the nucleotide composition of the mitogenomes are the basis of the rapid evolution of Chalcidoidea, which leads to inconsistent topologies based on different data types and analysis strategies (Wu et al. 2020). Currently, the CAT + GTR model implemented in PhyloBayes software was found to be the best fitting model for all data sets. In addition, the AA data set is considered as the best fit matrix for reconstructing phylogenetic trees (Li et al. 2015, 2017).

In the BI topology based on the AA data set, *A.arizonensis* and *M.eriococci* formed a clade representing Tetracneminae. The remaining six species formed a monophyletic clade representing Encyrtinae. In Encyrtinae, *Encyrtus* formed a monophyletic clade as a sister group to the clade formed by *O.plautus* and *D.aligarhensis*. *Encyrtussasakii* and *E.rhodooccisiae* are most closely related in this monophyletic clade. In the BI topology based on the PCG data set, *E.eulecaniumiae* and *E.sasakii* form a sister group then to *E.rhodooccisiae*. *Encyrtusinfelix* was the first diverged in *Encyrtus*.

**Figure 9.**
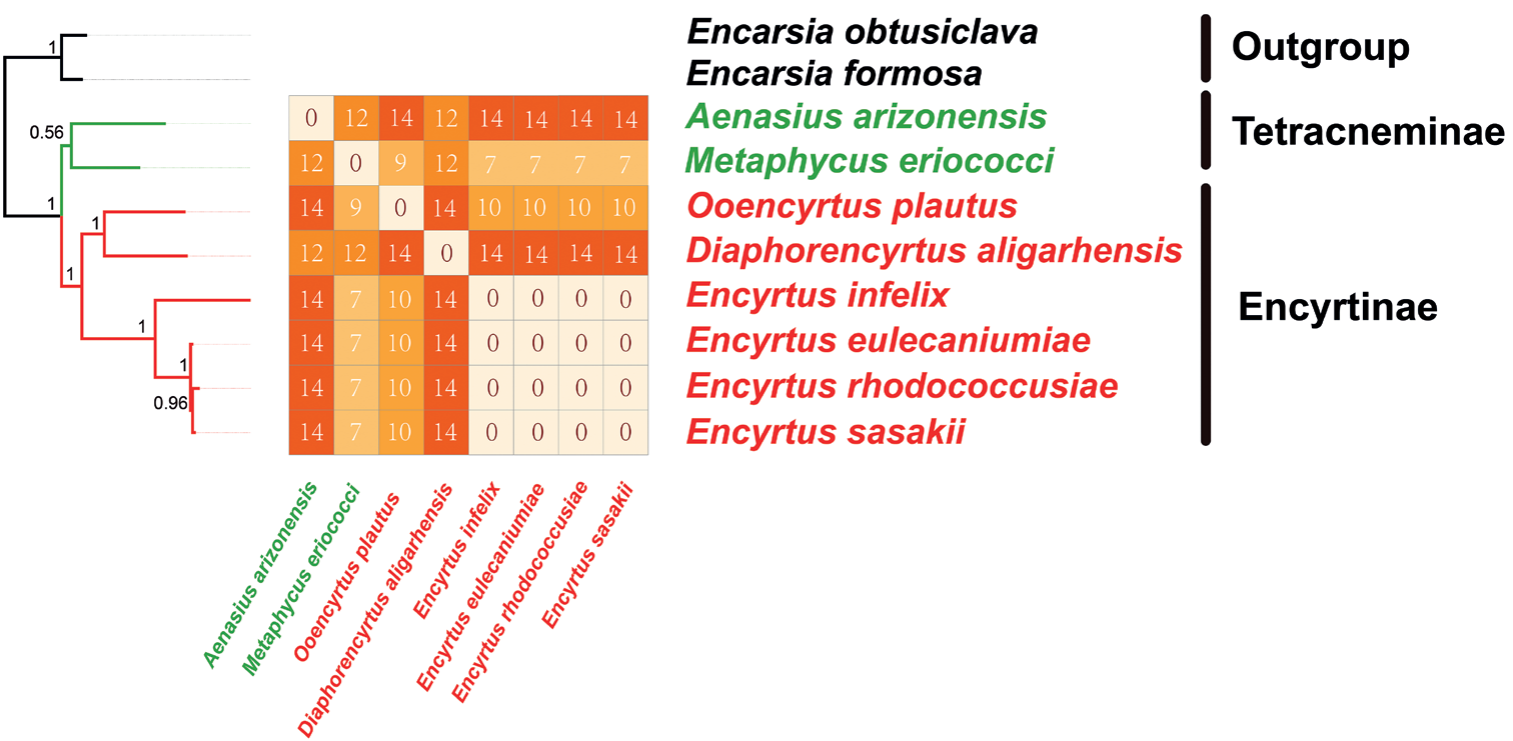
Mitogenomic phylogeny of eight Encyrtidae species and two outgroups based on the AA data set using the Bayesian inference. Heatmap of PBD values among encyrtid species is nested within the phylogeny, and the PBD values are listed.

Pairwise breakpoint distances (PBD) between the mitochondrial genomes of each species in Encyrtidae were calculated using the web server CREx, and heatmaps were constructed (Fig. 9). In the BI topology based on the AA data set, *E.infelix* was the first divergence branch in *Encytus*, which is consistent with the structure of the rest phylogenetic trees. The value of pairwise breakpoint distances between *E.infelix* and *D.aligarhensis* was 14, and the value of *E.infelix* and *O.plautus* was 10. Lower values of PBD indicated closer relationships which was consistent with the topology among *E.infelix*, *O.plautus*, and *D.aligarhensis* on the phylogenetic tree.

## ﻿Discussion

In Encyrtidae, the gene rearrangement processes can be summarized into four rearrangement mechanisms, which can also be applied to Chalcidoidea (Fig. 2). First, the rearrangement between *trnI-nad2-trnW* includes the inversion and translocation. The gene cluster *trnI-nad2-trnW* presents three gene orders, one is *trnI-nad2-trnW*, another is *trnW-trnI-nad2*, and the last is one of them has a translocation. For example, in Pteromalidae, *nad2* in *Sycobia* sp. was translocated, but in other species, the order of *trnI-nad2-trnW* has not changed. The second is the rearrangement in gene cluster *trnN-trnS1-trnY-trnC-trnR* where gene rearrangements occurred most frequently. The main rearrangements were inversions and long-distance translocations (mainly *trnR*). In *Encyrtus*, translocation of *trnR* were occurred in all these four species, and this phenomenon was also found in Agaonidae. The final mechanism is the inversion of *trnE-trnF* and *trnP-trnT.* The *trnE-trnF* inversion occurred in *A.arizonensis*, *D.aligarhensis*, *Tamarixiaradiata*, and *Aisopteromalusclandrae*. And the *trnP-trnT* inversion occurred in *Ceratosolenfusiceps*, *Eupelmus* sp. and *A.arizonensis*.

These four gene rearrangements can be applied to most of Chalcidoidea. When these tRNA gene rearrangement patterns were mapped on the estimated phylogenetic tree, the gene order of mitochondrial genome may resolve some contentious evolutionary questions (Shen et al. 2019). Based on the gene rearrangements occurring in Encyrtidae and the pairwise breakpoint distances heatmap, the hypotheses can be verified: the closely related species tend to have similar mitochondrial gene orders (Wu et al. 2020). For example, the gene order of all species of *Encytus* are identical. Correspondingly, the value of pairwise breakpoint distances among them are zero. Besides, the long-distance translocation of *trnQ* occurred in *D.aligarhensis* and *O.plautus*. In the topology of the BI tree based on AA matrix, *D.aligarhensis* and *O.plautus* are closely related and sister to the clade formed by genus *Encytus*. The PBD value between *O.plautus* and *Encyrtus* is 10, and the PBD value between *D.aligarhensis* and *Encyrtus* is 14. Lower PBD values indicates that *O.plautus* is more closely related to *Encyrtus*, which is in accordance with the phylogenetic tree. This phenomenon verified that PBD values could be used for inferring phylogenetic relationships. In summary, the gene rearrangement of mitogenome can provide a valuable source of characteristics for the reconstruction of molecular phylogeny (Wu et al. 2020).

As the sampling diversity of Encyrtidae is limited, it cannot completely solve the main classification question. Considering the limited research on taxonomic relationships inference based on molecular data, a comprehensive comparison of species morphology and genetic characteristics is needed to better understand the phylogenetic relationship of Encyrtidae. If more mitochondrial genomes are sequenced, the accuracy of phylogenetic relationships could be improved. We hope that future studies will combine morphology with more data sets from the mitochondrial genome to provide sufficient evidence for the phylogenetic relationship of the Encyrtidae.

## ﻿Conclusions

In this study, we sequenced the nearly complete mitogenome of *O.plautus* that contains 37 genes and one control region. The nucleotide composition, codon usage, RNA structures, and protein-coding genes evolution were analyzed. The mitogenome genome of *O.plautus* reveals the phylogenetic relationship of Encyrtidae for the first time. In addition, the regularity of gene rearrangement within Encyrtidae is discussed. The results of this study will contribute to further studies on evolutionary relationships within Encyrtidae.

## ﻿Funding

This work was supported by the National Natural Science Foundation of China (grant no. 32100352, 32100355), the Natural Science Fund of Anhui Province (grant no. 1908085QC93), the Natural Science Foundation of Universities of Anhui Province (grant no. KJ2020A0094), the Major Science and Technology Projects in Anhui Province (grant no. 202003a06020009), the National Science and Technology Fundamental Resources Investigation Program of China (grant no. 2019FY101800) and the Natural Science Foundation of Anhui Normal University (grant no. 2020XJ19).
